# Complement C1q-mediated microglial synaptic elimination by enhancing desialylation underlies sevoflurane-induced developmental neurotoxicity

**DOI:** 10.1186/s13578-024-01223-7

**Published:** 2024-04-01

**Authors:** Gang Wang, Hua-yue Liu, Xiao-wen Meng, Ying Chen, Wei-ming Zhao, Wen-ting Li, Han-bing Xu, Ke Peng, Fu-hai Ji

**Affiliations:** 1 Department of Anesthesiology, First Affiliated Hospital of Soochow University, 188 Shizi Street, Suzhou, 215006 Jiangsu China; 2 Institute of Anesthesiology, Soochow University, Suzhou, 215006 Jiangsu China; 3 Ambulatory Surgery Center, First Affiliated Hospital of Soochow University, Suzhou, 215006 Jiangsu China; 4 Departments of Neurology, First Affiliated Hospital of Soochow University, Suzhou, 215006 Jiangsu China

**Keywords:** Sevoflurane, Neonatal, Microglia, Synapse, Complement C1q, Sialic acid, Neurotoxicity

## Abstract

**Background:**

Repeated neonatal sevoflurane exposures led to neurocognitive disorders in young mice. We aimed to assess the role of microglia and complement C1q in sevoflurane-induced neurotoxicity and explore the underlying mechanisms.

**Methods:**

Neonatal mice were treated with sevoflurane on postnatal days 6, 8, and 10, and the Morris water maze was performed to assess cognitive functions. For mechanistic explorations, mice were treated with minocycline, C1q-antibody ANX005, and sialidase-inhibitor N-acetyl-2,3-dehydro-2-deoxyneuraminic acid (NADNA) before sevoflurane exposures. Western blotting, RT-qPCR, Golgi staining, 3D reconstruction and engulfment analysis, immunofluorescence, and microglial morphology analysis were performed. In vitro experiments were conducted in microglial cell line BV2 cells.

**Results:**

Repeated neonatal sevoflurane exposures resulted in deficiencies in learning and cognition of young mice, accompanied by microglial activation and synapse loss. Sevoflurane enhanced microglia-mediated synapse elimination through C1q binding to synapses. Inhibition of microglial activation and phagocytosis with minocycline significantly reduced the loss of synapses. We further revealed the involvement of neuronal sialic acids in this process. The enhanced activity of sialidase by sevoflurane led to the loss of sialic acids, which facilitated C1q binding to synapses. Inhibition of C1q with ANX005 or inhibition of sialidase with NADNA significantly rescued microglia-mediated synapse loss and improved neurocognitive function. Sevoflurane enhanced the engulfment of BV2 cells, which was reversed by ANX005.

**Conclusions:**

Our findings demonstrated that C1q-mediated microglial synaptic elimination by enhancing desialylation contributed to sevoflurane-induced developmental neurotoxicity. Inhibition of C1q or sialidase may be a potential therapeutic strategy for this neurotoxicity.

**Supplementary Information:**

The online version contains supplementary material available at 10.1186/s13578-024-01223-7.

## Background

The early stage after birth is a pivotal time window of neurodevelopment. There are growing concerns that prolonged or repeated anesthesia exposures during this period may lead to adverse learning and memory outcomes in later life, such as poor academic performance and language disability [1–4]. Sevoflurane is the most commonly used anesthetic agent for pediatrics [5]. Recent studies have identified its potential to induce developmental neurotoxicity. In animal models, prenatal and neonatal exposures to sevoflurane caused neuronal apoptosis, neuroinflammation, neurogenesis impairment, synapse loss, and dysfunction of synaptic plasticity [6–10]. Sevoflurane-induced neurotoxicity is closely related to the hippocampus, a brain area that plays a critical role in cognition [11]. Sevoflurane-treated mice exhibited neurocognitive impairment, alterations in hippocampal synaptic transmission, synapse loss, and dendritic damage [12–14]. However, the mechanism of sevoflurane-induced neurotoxicity is not fully understood.

Microglia, the resident immune cells in the central nervous system, constantly survey the brain to remove redundant synapses, debris, or dying cells through phagocytosis [15]. Neurons form exuberant synapses in the postnatal period, and microglia play an important role in synapse pruning [16, 17]. Additionally, synaptic phagocytosis by microglia is involved in the pathogenesis of many diseases such as autism, Alzheimer’s disease, and demyelinating diseases [18–20]. The complement system plays a crucial role in the developmental process of synaptic pruning by microglia [21, 22]. C1q is the initiating protein in the complement cascade. A recent study showed that prolonged sevoflurane exposure increased C1q expression in the rat hippocampus, and deletion of C1q prevented microglial synaptic engulfment to alleviate cognitive dysfunction [23]. Several factors affect the binding of C1q to synapses, such as CD47, neuronal pentraxins, and sialic acids [24]. The removal of sialic acid (termed desialylation) by sialidases on neurons encourages synapse tagging by C1q, thereby promoting synapse pruning [25, 26].

In this study, we investigated the effects of repeated neonatal exposures to sevoflurane on neurocognitive function in young mice and the role of C1q-mediated synaptic engulfment by microglia. We hypothesized that sevoflurane exposures activated microglia in the hippocampus, promoted C1q binding to synapses via desialylation, and thus enhanced microglial synaptic elimination, which is a key mechanism underlying sevoflurane-induced neurotoxicity.

## Methods

### Ethics and animals

This study was approved by the Institutional Animal Care and Use Committee of Soochow University (Approval No. 202210A0168). C57BL/6 J mice (20–25 g) were obtained from the Cavens Laboratory (Changzhou China) and fed standard diets under a controlled environment (12 h light–dark cycle, room temperature at 20–22 °C, and relative humidity of 60%). Male offspring mice were used in the experiments. All procedures complied with the Guide for the Care and Use of Laboratory Animals published by the US National Institutes of Health (NIH Publication No. 85–23, revised in 1996).

The sample size was determined based on recent literature (Western blot and real-time quantitative polymerase chain reaction [RT‒qPCR], n = 3–6; immunostaining, n = 6; behavioral test, n = 8) [10]. Randomization was conducted with the use of a computer-generated table of random numbers. Neonatal mice sequentially numbered with ear tags were randomly allocated into different groups. The allocation list was sealed using opaque envelopes. The researchers were blinded to the group allocation while performing the behavior tests, immunofluorescence, Western blot, and RT‒qPCR during this study.

### Repeated neonatal sevoflurane exposures

The neonatal mice were randomly divided into the control group or the sevoflurane group. On postnatal days (PNDs) 6, 8, and 10, mice in the sevoflurane group were placed in a closed chamber. Using the Datex-Ohmeda anesthesia system (Madison, WI, USA), mice received 3% sevoflurane mixed with 60% oxygen for 2 h, with a flow of 2 L/min for anesthesia induction, followed by 1 L/min for maintenance. The control group received 60% oxygen without sevoflurane for 2 h. The concentrations of sevoflurane and oxygen were continuously monitored (Vamos; Dräger Medical, Germany), and the mice were kept at 37 ± 1 °C on a warming pad throughout the procedures. After sevoflurane treatment, the mice were immediately placed back into their home cages and received standard care. The characteristics of weight, blood gas, and electrolytes of mice are listed in Additional file 2: Table S1.

### Cell culture and sevoflurane treatment

Microglial cell line BV2 cells were obtained from Fu Heng Biology (Shanghai, China). The cells were cultured in Dulbecco’s modified eagle medium containing 10% (v/v) fetal bovine serum (FBS) (Gibco, Germany) and 1% (v/v) penicillin/streptomycin at 37 ℃ and 5% CO_2_ humidified atmosphere. The cells were cultured for 24 h, and then the medium was renewed. To establish the sevoflurane-treated in vitro model, the cells were treated with 3% sevoflurane solution (dissolved in Methyl sulfoxide) for 6 h [27].

### Morris water maze (MWM) test

The cognitive performance of mice was assessed using the MWM test on PNDs 31–36. The test was performed by a skilled operator blinded to the group assignment. The round steel pool was 120 cm in diameter and 60 cm in height, which was filled with opaque water (21 ℃) using titanium dioxide. The pool was divided into four equal quadrants.

At first, we performed the visible platform trial to assess whether the swimming ability and vision of the mice were normal [28]. Briefly, the platform (10 cm in diameter, 30 cm in depth) with a back flag on it was placed 1 cm above the water surface, so that the mice could see the platform. The mice were released gently into the water facing the wall from different quadrants, and allowed to find the platform. The times to find the platform and the swimming speed were recorded using a video tracking system (XR-XM101, Shanghai Xinruan Information Technology Co., Ltd).

Next, we performed the hidden platform trial in which the same platform without a flag was placed 1 cm below the water surface. In the training phase on PNDs 31–35, mice were trained to reach the platform for 5 consecutive days with 4 trials per day, and the intertrial interval was at least 60 min. At the beginning of each trial, mice were placed into the water facing the wall from all 4 quadrants and allowed to find the hidden platform in 60 s and stay on it for 15 s. If the animal failed to locate the platform within 60 s, it was gently guided to stay on the platform for 15 s. On PND 36, the platform was removed, and the mice were placed in the quadrant opposite to the platform and allowed to swim freely for 60 s. The escape latency, platform cross times, time spent in the target quadrant, and swimming speed were measured.

On the first day of training (PND 31), most mice (75/80, 93.75%) failed to locate the hidden platform within 60 s, and they were guided to stay on the platform for 15 s. On the third day of training (PND 33), only 1 mouse (1/80, 1.25%) failed to locate the hidden platform within 60 s, and it was guided to the platform. On the last two days of training (PNDs 34–35), all mice could locate the hidden platform within 60 s. Thus, all mice completed the training phase and included in the statistical analysis.

### Drug administration

Mice were treated with minocycline (Sangon Biotech Inc, China) 75 mg/kg, C1q-antibody ANX005 (Annexon Biosciences) 20 mg/kg, or N-acetyl-2,3-dehydro-2-deoxyneuraminic acid (NADNA, Sigma-Aldrich, St Louis, MO, USA) 10 mg/kg by intraperitoneal injection in a 50 μl volume 30 min before sevoflurane exposures. Phosphate-buffered saline (PBS) or IgG was used as a control. Minocycline has been shown to inhibit microglial activation and engulfment of synapses in vitro and in vivo. NADNA was used to inhibit the activity of sialidase.

### Western blot analysis

After behavioral testing on PND 36, mice were anesthetized with 3% sevoflurane and the hippocampus was harvested for further experiments. The hippocampus of mice or cultured cells were lysed on ice for 30 min with radioimmunoprecipitation lysis buffer (Beyotime, Jiangsu, China) containing a cocktail of protease inhibitors. Protein concentration was quantified using the Bicinchoninic acid assay (Beyotime, Jiangsu, China). Protein samples were separated by 10% gel in sodium dodecyl sulfate–polyacrylamide gel electrophoresis (SDS‒PAGE), and proteins were transferred to the Polyvinylidene fluoride (PVDF) membranes. The membranes were blocked with Tris-buffered saline and tween (TBST) containing 5% nonfat milk and 0.1% Tween 20 for 2 h at room temperature, and then incubated with the following primary antibodies overnight at 4 °C: rabbit anti-PSD95 (1:1000; ZEN BIO, 381,001), rabbit anti-Vglut2 (1:1000; Affinity Biosciences, DF13296), rabbit anti-C1qa (1:1000; Absin, abs137316), rabbit anti-C3 (1:1000; Proteintech, 21337–1-AP), and mouse anti-β-tubulin (1:1000; Beyotime, AF2839). The membranes were then incubated with HRP-labeled goat anti-mouse (1:2000; Beyotime, A0216) and goat anti-rabbit (1:2000; Beyotime, A0208) secondary antibodies for 2 h at room temperature. The bands were detected by Tanon 5200 (Tanon Science & Technology Co. Ltd) and analyzed using ImageJ software (NIH). Densitometric values of the proteins were normalized to β-tubulin.

### Differentially expressed genes (DEGs)

The raw RNA data were acquired from the National Center for Biotechnology Information (https://www.ncbi.nlm.nih.gov/search/all/?term=PRJNA556843) and analyzed as previously reported by Song et al. [10]. The raw p values were adjusted with the use of the false discovery rate (FDR). The cutoff criteria for DEG screening included FDR < 0.05 and ǀlog_2_ fold change (FC)ǀ > 1.

### RT‒qPCR

The hippocampal tissues were subjected to RNA extraction, reverse transcription, and RT-qPCR as previously described [10]. The following primers (Sangon Biotech Inc, China) were used in the process: mouse NEU1 (forward, 5′-GAT CGG CTC TGT AGA CAC TTT C-3′; reverse, 5′-CCC TCA TCG GAT GCA GAT TTT T-3′), mouse NEU2 (forward, 5′-AGT TGA TTG TCC TGA GAA GAG G-3′; reverse, 5′-CAG CGA TGA AGA AAA GGA AGA G-3′), mouse NEU3 (forward, 5′-TAC CTG TTT TTC ATC TGT GTG C-3′; reverse, 5′-CTC GGT CAA GTC TTT CAC TTC A-3′), mouse NEU4 (forward, 5′-ACT GGA GGA GCA CAG GTC TAT GAA C-3′; reverse, 5′ AGC ACG GCA ATG AAG AAG AGG AAG-3′), and mouse β-Tubulin (5′-CAG CGA TGA GCA CGG CAT AGA C; reverse, 5′-CCA GGT TCC AAG TCC ACC AGA ATG-3′).

### Golgi staining

Golgi staining was performed using the FD Rapid Golgi staining kit (PK401, FD NeuroTechnologies, United States) according to the manufacturer’s instructions. The brain samples were collected and immersed in a mixture of solutions A and B (1:1) in the dark at room temperature for 14 days. Thereafter, the samples were transferred to solution C in the dark for 5 days, embedded in optimal cutting temperature compound reagent, and frozen at − 80 ℃ for 24 h. The brains were cut into coronal Sects. (100 μm thick) using a cryostat microtome (Leica, Wetzlar, Germany). The staining procedure was performed following the manufacturer’s instructions. The slides were viewed using a light microscope with a 63 × oil-immersion objective lens (Olympus, FV1200). Pyramidal neurons in the CA1 region that were well-impregnated and clearly distinguishable from other neurons were analyzed. Five basal dendrite segments of 30 μm or longer were randomly selected from each pyramidal neuron. The dendritic spine density was analyzed using ImageJ.

### Three-dimensional reconstruction and engulfment analysis

Brain sections were scanned with a 63 × oil immersion objective using an Olympus FV1200 confocal microscope (Olympus, Japan) with a 0.5 μm z-step (z = 25–35 frames, 211 × 211 μm^2^ in area). Optical stacks were acquired in the CA1 region from 2-to-3 tissue sections per animal to reconstruct three-dimensional (3D) microglia. To analyze the engulfment of synapses by microglia and astrocytes, we performed a double immunostaining for the postsynaptic marker PSD95 with microglia (marker IBA1) or astrocytes (marker GFAP), and we performed 3D image rendering of z-stack images using Imaris software (version 9.0.1, Bitplane, Switzerland). Briefly, microglial/astrocyte cells and PSD95-positive synapses were reconstructed using the surface rendering function, and we colocalized the PSD95 volume within the IBA1/GFAP volume to produce a microglial/astrocyte synaptic engulfment volume. The engulfment index was calculated as the volume of internalized PSD95 divided by the volume of microglia/astrocytes.

### Engulfment analysis in vitro

For phagocytosis assay in vitro, microglial cell line BV2 cells (1 × 10^4^) were seeded in the confocal dish and cultured for 24 h. Then the medium was renewed, and the cells were treated with 3% sevoflurane solution (dissolved in Methyl sulfoxide) for 6 h [27]. Subsequently, the medium was discarded, and the cells were washed with phosphate buffer saline (PBS) and cultured in the new medium. The fluorescent latex beads (1 µm, L2778, Sigma) were pre-opsonized in 50% FBS and PBS, and the beads were added to the BV2 cells at a concentration of 50 beads per BV2 cell for incubation at 37 °C for 6 h. After incubation, the cells were washed gently to remove the remaining beads, and the cells were fixed by 4% paraformaldehyde (PFA). Images were acquired with an Olympus FV1200 confocal microscope (Olympus, Japan) using a 63 × /1.3 NA oil objective.

### Immunofluorescence

Mice were anesthetized and perfused with 30 ml 1 × PBS followed by 30 ml 4% PFA, and then the brains were harvested. The brains were postfixed with 4% PFA at 4 ℃ for 3 h and cryoprotected with 15 and 30% sucrose at 4 ℃ for 24 h. Serial coronal Sects. (20 μm) were prepared using a cryostat microtome (Leica CM1950). The brain sections were washed in PBS for 3 × 10 min and then incubated in a blocking solution (5% bull serum albumin and 0.1% Triton X-100 in PBS) at room temperature for 2 h. Subsequently, the sections were incubated with primary antibodies at 4 ℃ overnight, including PSD95 (1/200, 381001, ZEN BIO, China), PSD95 (1/200, ab12093, Abcam, UK), Vglut2 (1/200, NBP2-59330, NOVUS, USA), C1q (1/200, DF7839, Affinity, China), C1q (1/200, 67063–1, Proteintech, USA), IBA1 (1/400, ab283342, Abcam, UK), GFAP (1/200, AF1177, Beyotime), CD68 (1/200, 29176, CST, USA), CD11b (1/200, R380675, ZEN BIO, China), MAP2 (1/200, 67015–1, Proteintech, USA), polysialic acid neural cell adhesion molecule (PSA-NCAM) (1/200, 14–9118-82, Invitrogen, USA), and peanut agglutinin (PNA)-FITC (1/1000, L7381, Sigma, USA). The next day, the brain sections were washed with PBS for 3 × 10 min followed by incubation with secondary antibodies at room temperature for 2 h, including Alexa Fluor 488 (1/200, A0423, Beyotime), Alexa Fluor 488 (1/200, A-11055, Invitrogen), Alexa Fluor 647 (1/200, A0468, Beyotime), Cy3 (1/200, A0521, Beyotime), and Hoechst 33342 (1/10000, C1022, Beyotime). Finally, images were acquired using the Olympus FV1200 confocal microscope and analyzed using ImageJ.

### Analysis of microglial morphology

Z-stack images were acquired at 0.5 μm intervals over a 15 μm Z-range using the Olympus FV1200 confocal microscope. The Z-stack images were converted into single-plane maximal intensity projections. Cell morphology was analyzed using the AnalyzeSkeleton plugin in ImageJ. Briefly, a thresholding method was applied to binarize the images. Unsharp mask and despeckle filters were applied to remove noise. Finally, the skeletonized images were analyzed.

### Statistical analyses

GraphPad Prism 9 Software was used for all statistical analyses. The normality of the data was checked with the Shapiro–Wilk test, and the data are presented as mean ± standard error of the mean (SEM). Two groups were compared using an unpaired t-test; comparisons among four groups were analyzed with one-way or two-way ANOVA, followed by Tukey’s post-hoc test for multiple comparisons. Correlation analysis was conducted using Pearson’s correlation coefficient. A two-tailed *P* value < 0.05 was considered statistically significant.

## Results

### Repeated neonatal sevoflurane exposures induced cognitive dysfunction and hippocampal synapse loss in young mice

**Fig. 1:**
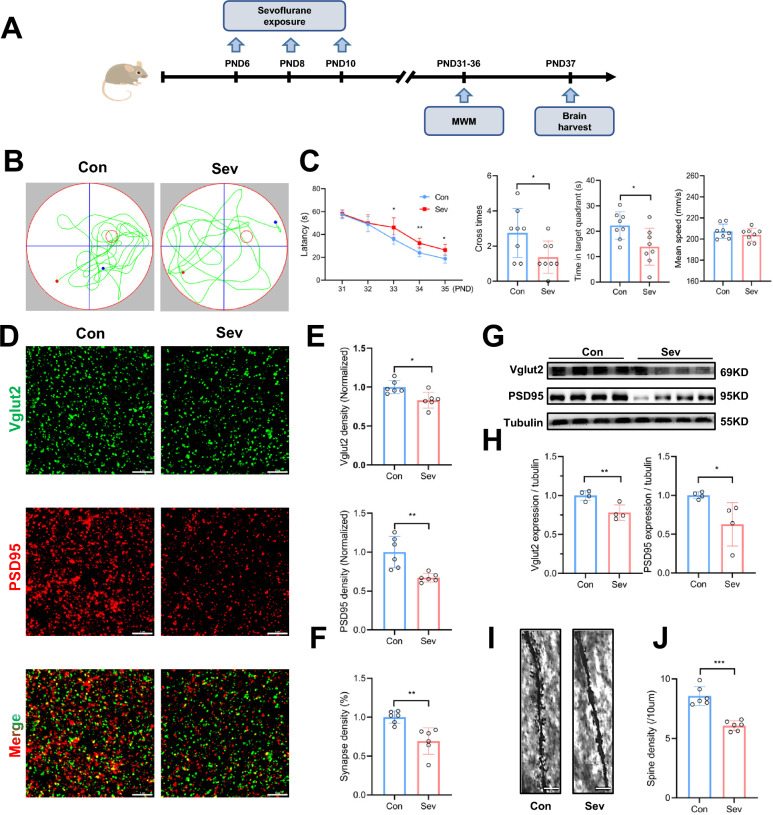
Cognitive dysfunction and synapse loss in the hippocampus of mice after neonatal sevoflurane exposures. **A** Scheme of neonatal mice receiving repeated sevoflurane and the MWM test. Neonatal mice were exposed to 3% sevoflurane on PNDs 6, 8, and 10, and then the memory and learning abilities were assessed using the MWM test on PNDs 31–36. **B** Tracking plots of mice. **C** Sevoflurane reduced the escape latency compared to the control group on PNDs 33–35, as well as the platform crossing times and times spent in the target quadrant. Swimming speed was similar between groups. n = 8. Unpaired t-test and two-way ANOVA. **D** Representative confocal microscopy images displaying the immunoreactivity of the presynaptic marker Vglut2 (green) and postsynaptic marker PSD95 (red) in the hippocampus. Scale bar = 5 μm. **E** The sevoflurane group showed decreased density of Vglut2 and PSD95. n = 6. Unpaired t-test. **F** Colocalization analysis showed that the density of synapses was lower in sevoflurane-treated mice. n = 6. Unpaired t-test. **G** Representative Western blot bands of Vglut2 and PSD95 in the two groups. (**H**) Quantification of Western blot showed that the expression of Vglut2 and PSD95 was decreased in sevoflurane-treated mice. n = 4. Unpaired t-test. **I** Representative Golgi-Cox staining images of dendritic spines in the hippocampus. Scale bar = 5 μm. **J** Quantification of the density of dendritic spines showed that the sevoflurane group had a lower spine density. n = 6. Unpaired t-test. Data are mean ± SEM. *P < 0.05, ** P < 0.01, *** P < 0.001. MWM, Morris water maze; PND, postnatal day

To investigate the long-term effects of repeated neonatal sevoflurane anesthesia on cognitive function in young mice, neonatal mice were exposed to 3% sevoflurane with 60% oxygen for 2 h on PNDs 6, 8, and 10 (Fig. 1A). Spatial learning and memory ability was measured by the MWM test on PNDs 31–36 (Fig. 1B). The results showed significant main effects of time including days 31–35 [F (_4, 70_) = 121.3, *P* < 0.001] and interventions of control and sevoflurane [F (_1, 70_) = 22.45, *P* < 0.001] (Fig. 1C). The sevoflurane-treated mice spent more time finding the platform on PNDs 33–35. Compared to the control mice, sevoflurane-treated mice had significantly fewer platform cross times (1.38 ± 0.92 vs. 2.75 ± 1.38, *P* = 0.035) and less time in the target quadrant (13.90 ± 7.24 vs. 22.24 ± 5.45 s, *P* = 0.021) on PND 36, without a between-group difference in the mean swimming speed (Fig. 1C).

To assess the effect of sevoflurane on synapses, we performed immunofluorescence and immunoblotting to assess the expression of the presynaptic marker Vglut2 and the postsynaptic marker PSD95. We found decreased fluorescence density of Vglut2 (*P* = 0.011) and PSD95 (*P* = 0.003) in sevoflurane-treated mice (Fig. 1D, E). Furthermore, the quantification of colocalized pre- and postsynaptic puncta (Vglut2^+^ and PSD95^+^) revealed a significant loss of synapses (*P* = 0.002) (Fig. 1F). The Western blot results also showed that sevoflurane downregulated the protein expression of Vglut2 and PSD95 in the hippocampus (Fig. 1G, H). We further examined the dendrite spine density by Golgi staining, showing that sevoflurane significantly reduced the number of pyramidal neuron spines located at the stratum radiatum in the CA1 region (*P* < 0.001) (Fig. 1I, J).

Taken together, these data indicated that repeated sevoflurane exposures during the neonatal period led to the loss of synapses and deleterious effects on long-term learning and memory function in young mice.

### Repeated neonatal sevoflurane exposures resulted in synapse loss by excessive microglial phagocytosis in the hippocampus

**Fig. 2:**
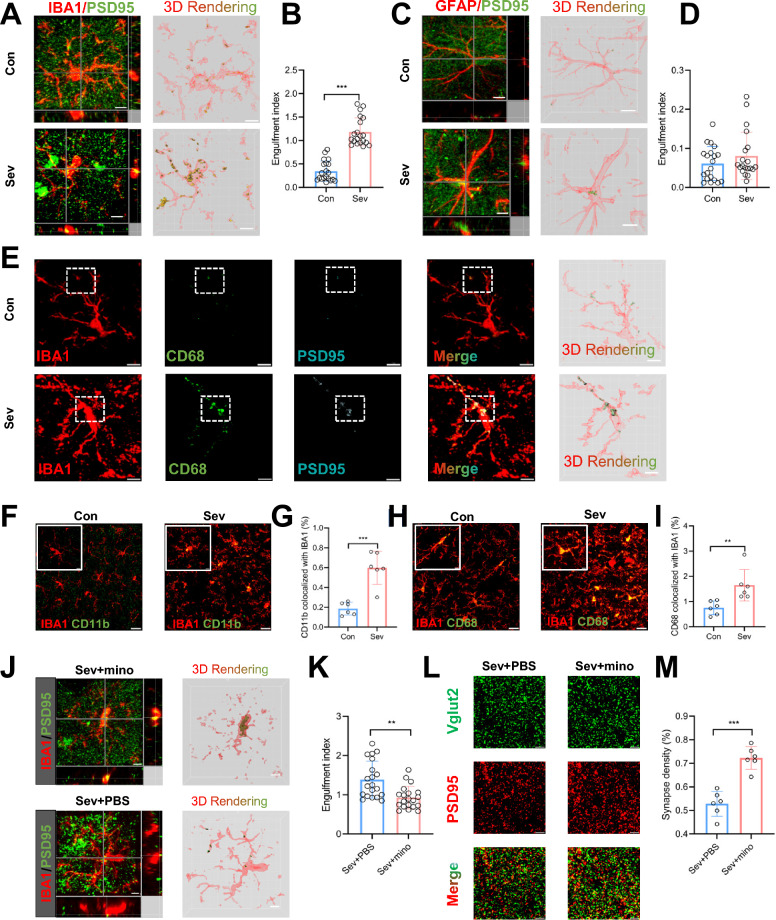
Synapse loss caused by excessive microglial phagocytosis in the hippocampus of mice after neonatal sevoflurane exposures. **A** Representative confocal images of IBA1^+^ microglia (red) containing PSD95^+^ puncta (green). Orthographic view and 3D rendering are shown. Scale bars = 5 μm. **B** Quantification analysis showed that the sevoflurane group had increased PSD95^+^ puncta in microglia. n = 20. Unpaired t-test. **C** Representative confocal images of GFAP^+^ astrocytes (red) containing PSD95^+^ puncta (green). Orthographic view and 3D rendering are shown. Scale bars = 5 μm. **D** Quantification analysis showed that the two groups had similar volumes of PSD95^+^ puncta in astrocytes. n = 20. Unpaired t-test. **E** Representative confocal images and 3D surface rendering of PSD95^+^ puncta (cyan) in CD68^+^ (green) positive microglia (red). Scale bars = 5 μm. **F** Representative confocal images of CD11b (green) and IBA1 (red) immunoreactivity. Scale bars = 20 μm. **G** Quantification analysis showed that the sevoflurane group had increased colocalization of CD11b and IBA1. n = 6. Unpaired t-test. **H** Representative confocal images of CD68 (green) and IBA1 (red) immunoreactivity. Scale bars = 20 μm. **I** Quantification analysis showed that the sevoflurane group had increased colocalization of CD68 and IBA1. n = 6. Unpaired t-test. **J** Representative confocal images and 3D surface rendering of IBA1^+^ microglia (red) containing PSD95^+^ puncta (green) in sevoflurane-treated mice receiving PBS or minocycline. Scale bars = 5 μm. **K** Quantification showed that minocycline led to a decrease in the engulfment index. n = 20. Unpaired t-test. **L** Representative confocal images of Vglut2 (green) and PSD95 (red) in sevoflurane-treated mice receiving PBS or minocycline. Scale bars = 5 μm. **M** Quantification of colocalization revealed that minocycline increased synapse density. n = 6. Unpaired t-test. Data are mean ± SEM. *P < 0.05, ** P < 0.01, *** P < 0.001

Accumulating evidence suggests that glial cells participate in sculpting neuronal circuits through synapse engulfment [29]. To investigate whether microglia (marker IBA1) and astrocytes (marker GFAP) were involved in our model, 3D rendering was performed to assess engulfment. In sevoflurane-treated mice, we observed significantly increased PSD95^+^ puncta within microglia, indicating that microglia engulfed synapses (*P* < 0.001) (Fig. 2A, B). In contrast, no between-group difference was detected in the PSD95^+^ puncta within astrocytes (*P* = 0.246) (Fig. 2C, D). We also found that, compared to astrocytes, microglia eliminated more synapses in sevoflurane-treated mice (*P* < 0.001) (Additional file 1: Fig. S1A, B). In the in vitro engulfment assay, sevoflurane promoted microglia cell line BV2 cells phagocyted more fluorescent latex beads than in the control condition (Additional file 1: Fig. S1C), which was in line with in vivo results. Since lysosomes (marker CD68) are the main organelles that degrade proteins, apoptotic cells, and pathogens, we confirmed that the puncta engulfed in microglial lysosomes (PSD95^+^, IBA1^+^, and CD68^+^) were increased in sevoflurane-treated mice (Fig. 2E).

Next, we tested whether sevoflurane activated microglia and enhanced their phagocytic ability. Immunofluorescence analyses showed that sevoflurane significantly increased IBA1^+^ cell intensity (*P* < 0.001) (Additional file 1: Fig. S1D, E) and soma size (*P* < 0.001), and reduced the total branch points (*P* < 0.001) and total process length (*P* < 0.001) (Additional file 1: Fig. S1F, G). We further validated the activation and phagocytosis of microglia by co-staining IBA1 with CD11b or CD68, showing that the sevoflurane group had a significantly increased colocalization of IBA1 and CD11b (*P* < 0.001) (Fig. 2F, G) as well as IBA1 and CD68 (*P* = 0.002) (Fig. 2H, I), suggesting that sevoflurane enhanced microglial activation and phagocytic ability.

Based on these observations, we investigated whether the inhibition of microglial activation and phagocytosis by minocycline could rescue synapse loss in sevoflurane-treated mice. The immunostaining results showed that minocycline significantly reduced microglial phagocytosis of synapses, as reflected by decreased PSD95^+^ puncta within microglia (*P* = 0.003) (Fig. 2J, K), while minocycline had no significant effect on control mice (Additional file 1: Fig. S2A, B). We also found increased synapse density (colocalization of Vglut2 and PSD95) after minocycline administration in sevoflurane-treated mice (*P* < 0.001) (Fig. 2L, M), but not in control mice (Additional file 1: Fig. S2C, D).

Taken together, these data revealed that sevoflurane exposures activated microglia, enhanced microglial phagocytosis of synapses, and thus resulted in synapse loss, which was rescued by minocycline through inhibition of microglial activation and phagocytosis.

### Repeated neonatal sevoflurane exposures increased the expression of C1q in microglia in the hippocampal CA1 region

**Fig. 3:**
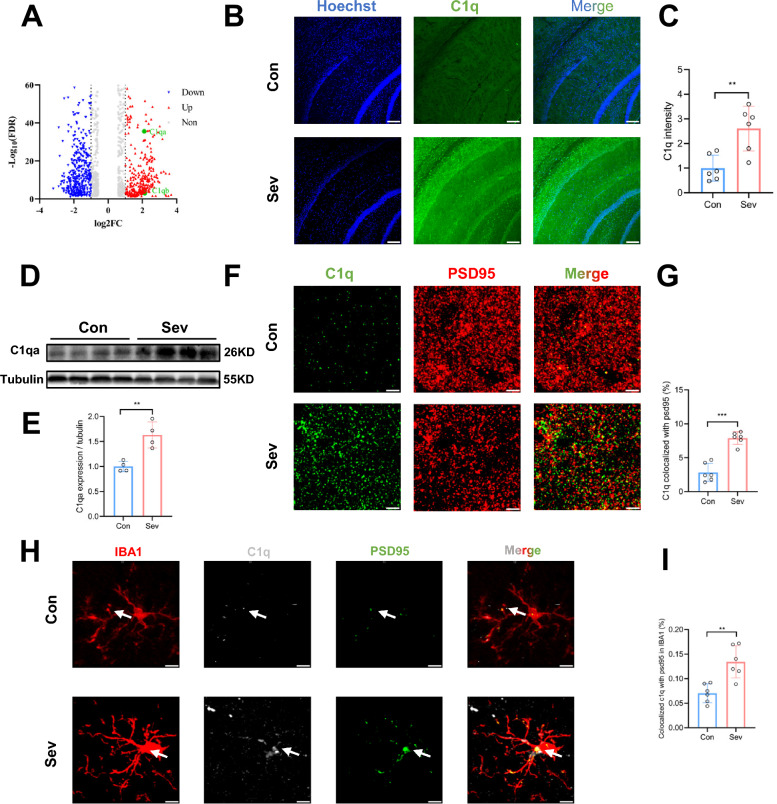
Increased expression of C1q in microglia of mouse hippocampus after neonatal sevoflurane exposures. **A** Volcano plot showing differentially expressed genes (ǀlog_2_FCǀ > 1, and FDR < 0.05), including C1qa and C1qb. **B** Representative confocal images of C1q puncta (green) and nuclei (blue) in the CA1 region. Scale bars = 100 μm. **C** Quantification showed that sevoflurane increased the expression of C1q. n = 6. Unpaired t-test. **D** Representative Western blot bands of C1qa in the CA1 region. **E** Western blot analysis showed that sevoflurane increased the level of C1qa. n = 4. Unpaired t-test. **F** Representative confocal images of C1q (green) and PSD95 (red) in the CA1 region. Scale bars = 5 μm. **G** Colocalization analysis revealed that C1q colocalized with PSD95 was elevated in sevoflurane-treated mice. n = 6. Unpaired t-test. **H** Representative confocal images of C1q (gray)-labeled PSD95 (green) within microglia (red). Scale bars = 5 μm. **I** Colocalization analysis showed that sevoflurane led to increased colocalization of C1q, PSD95, and IBA1. n = 6. Unpaired t-test. Data are mean ± SEM. *P < 0.05, ** P < 0.01, *** P < 0.001

From a mechanistic insight into sevoflurane-induced synaptic loss associated with microglia, we analyzed the DEGs in the hippocampus between sevoflurane-treated and control mice. Among the upregulated genes, C1qa and C1qb were closely related to microglial phagocytosis [30] (Fig. 3A). Immunofluorescence results showed that the intensity of C1q was significantly increased in the hippocampal CA1 region of sevoflurane-treated mice (*P* = 0.003) (Fig. 3B, C). Western blot analysis also confirmed the elevation of C1qa protein expression (*P* = 0.004) (Fig. 3D, E). In BV2 cells, the protein expression of C1q was significantly increased after sevoflurane exposure (Additional file 1: Fig. S3A, B).

C1q was colocalized with the microglial marker IBA1, but not the astrocyte marker GFAP, which indicated that C1q was mainly expressed in microglia (Additional file 1: Fig. S4A, B). In sevoflurane-treated mice, the increased C1q-positive puncta were colocalized with synaptic PSD95 (*P* < 0.001) (Fig. 3F, G), and the number of C1q-tagged PSD95^+^ puncta within the microglia was increased compared with that in the control mice (*P* = 0.002) (Fig. 3H, I). These data indicated that the activated microglia induced by neonatal sevoflurane exposures mainly engulfed C1q-binding synapses in the hippocampal CA1 region.

### Inhibition of C1q alleviated sevoflurane-induced cognitive dysfunction by decreasing microglia-mediated synapse loss in the hippocampus

**Fig. 4:**
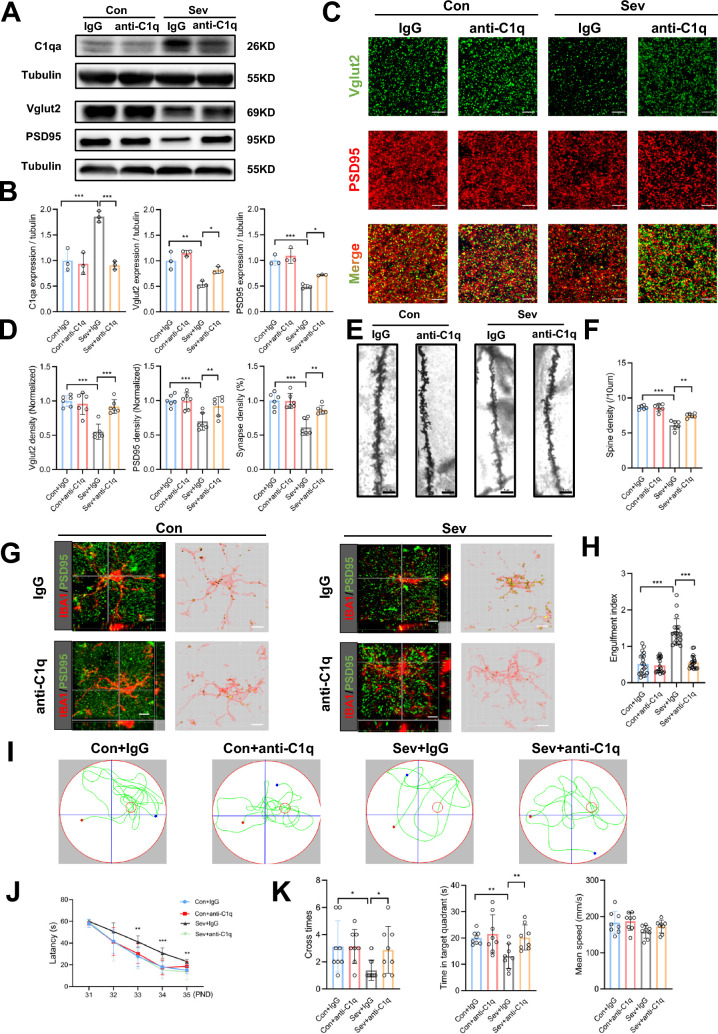
Alleviation of sevoflurane-induced cognitive dysfunction by C1q inhibition through decreasing microglia-mediated synapse loss. **A** Representative Western blot bands of C1qa, Vglut2, and PSD95 in the hippocampus of control and sevoflurane-treated mice with IgG or anti-C1q (ANX005) treatment. **B** Compared to treatment with sevoflurane and IgG, ANX005 downregulated C1q expression and upregulated Vglut2 and PSD95 expression. n = 3. One-way ANOVA followed by a post hoc Tukey’s test. **C** Representative confocal images of Vglut2 (green) and PSD95 (red) in the four groups. Scale bars = 5 μm. **D** Quantification analysis showed that ANX005 treatment increased the density of Vglut2^+^ puncta, PSD95^+^ puncta, and synapses compared to treatment with sevoflurane and IgG. n = 6. One-way ANOVA followed by a post hoc Tukey’s test. **E** Representative Golgi-Cox staining images of dendrite spines in the four groups. Scale bars = 5 μm. **F** Quantification analysis showed that ANX005 treatment increased the spine density compared to treatment with sevoflurane and IgG. n = 6. One-way ANOVA followed by a post hoc Tukey’s test. **G** Representative confocal images of IBA1^+^ (red) containing PSD95^+^ puncta (green) in the four groups. Orthographic view and 3D rendering are shown. Scale bars = 5 μm. **H** Quantification analysis showed that ANX005 treatment decreased the engulfment index compared to treatment with sevoflurane and IgG. n = 20. One-way ANOVA followed by a post hoc Tukey’s test. **I** Tracking plots of mice in the Morris water maze test in the four groups. **J** ANX005 treatment reduced the escape latency compared to treatment with sevoflurane and IgG. n = 8. Two-way RM ANOVA followed by a post hoc Tukey’s test. (**K**) ANX005 treatment increased platform cross times and time spent in the target quadrant compared to treatment with sevoflurane and IgG. n = 8. One-way ANOVA followed by a post hoc Tukey’s test. Data are mean ± SEM. *P < 0.05, ** P < 0.01, *** P < 0.001

C1q, as the initiating protein of the classical complement cascade, plays a crucial role in synapse elimination; therefore, we inhibited C1q expression by administration of the C1q-antibody ANX005. The Western blot results showed that C1q protein expression was effectively reduced by ANX005, and the expression of PSD95 and Vglut2 was increased in sevoflurane-treated mice (Fig. 4A, B). Then we analyzed the expression of C3, a downstream complement of C1q. The results showed that sevoflurane exposure increased the protein expression of C3 (Additional file 1: Fig. S5A, B), and inhibition of C1q resulted in a significant reduction of C3 expression (Additional file 1: Fig. S5C, D).

To detect whether inhibition of C1q could rescue synapse loss, we performed double-staining of PSD95 and Vglut2 to show that the decrease in synapse density was reversed by ANX005 (*P* = 0.001) (Fig. 4C, D). We also found an increased dendritic spine density after ANX005 administration (*P* = 0.003) (Fig. 4E, F). Moreover, colocalization of IBA1 and PSD95 revealed that ANX005 significantly inhibited the sevoflurane-induced microglial engulfment of synapses (*P* < 0.001) (Fig. 4G, H). ANX005 did not have a significant impact on synapses in the control mice. To verify the role of C1q in BV2 cells, we treated the cells with C1q-antibody ANX005 0.1 mM or IgG. The results showed that C1q-antibody had no effects on BV2 cell phagocytosis of fluorescent latex beads in the control group; however, C1q-antibody significantly reduced the number of fluorescent latex beads phagocyted by BV2 cells in the sevoflurane group (Additional file 1: Fig. S6).

We ruled out possible damage to vision and swimming ability due to sevoflurane or other treatments (IgG and anti-C1q ANX005) that may interfere with the outcome of the MWM test (Additional file 1: Fig. S7A, B). Next, we employed the MWM test to assess learning and memory ability in control and sevoflurane-treated mice with IgG or ANX005 treatment (Fig. 4I). Compared with IgG, ANX005 significantly shortened the escape latency on PNDs 33–35 in sevoflurane-treated mice (28.31 ± 11.94 vs. 41.24 ± 5.39 s, *P* = 0.005 on day 33; 15.29 ± 7.76 vs. 30.78 ± 4.8 s, *P* < 0.001 on day 34; 13.53 ± 7.43 vs. 23.07 ± 1.95 s, *P* = 0.003 on day 35) (Fig. 4J). ANX005 also led to increased platform crossing times (2.88 ± 1.73 vs. 1.38 ± 0.74, *P* = 0.04) and more time in the target quadrant (20.17 ± 4.78 vs. 13.06 ± 4.72 s, *P* = 0.001), without a significant between-group difference in the mean swimming speed (Fig. 4K).

Taken together, these data suggested that neonatal sevoflurane exposures induced learning and memory impairment through C1q-mediated synapse loss, while inhibiting C1q expression attenuated cognitive dysfunction.

### Repeated neonatal sevoflurane exposures induced desialylation of hippocampal neurons to facilitate C1q tagging to synapses in the hippocampal CA1 region

**Fig. 5:**
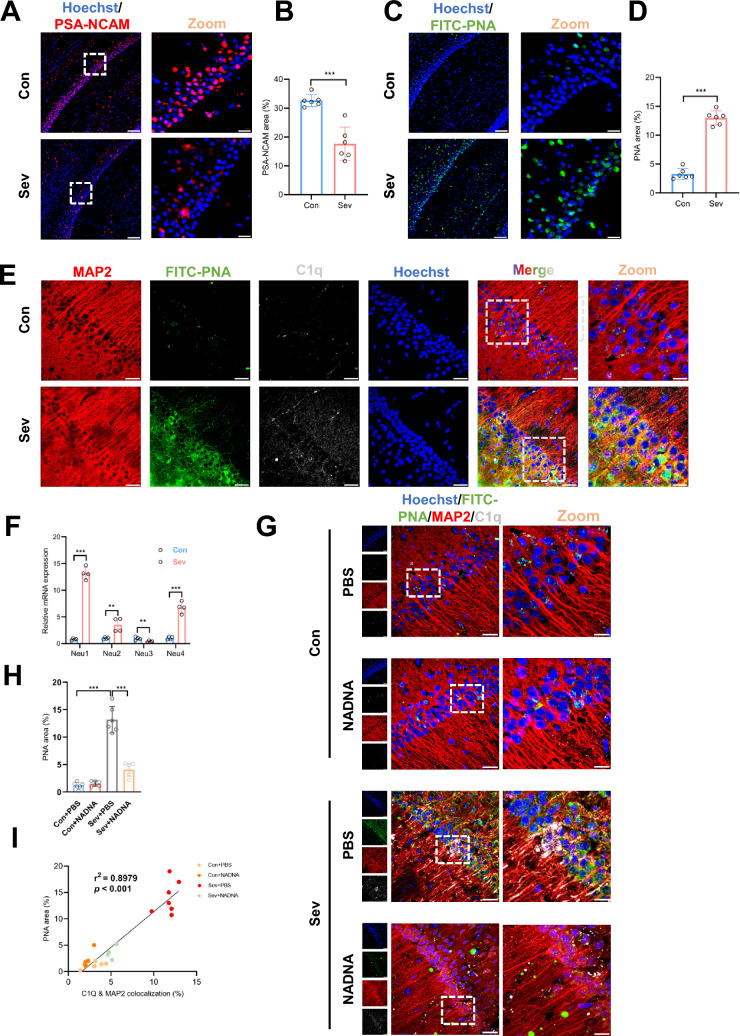
Increased C1q tagging to synapses caused by desialylation of hippocampal neurons after neonatal sevoflurane exposures. **A** Representative confocal immunostaining images of PSA-NCAM (red) and nuclei (blue) in the CA1 region of control and sevoflurane-treated mice. Scale bars = 100 μm, Scale bars = 20 μm. **B** Quantitative analysis showed that sevoflurane reduced sialic acids in the mouse hippocampus. n = 6. Unpaired t-test. **C** Representative confocal immunostaining images of FITC-PNA (green) and nuclei (blue) in the CA1 region of control and sevoflurane-treated mice. Scale bars = 100 μm, Scale bars = 20 μm. **D** Quantitative analysis showed that sevoflurane increased desialylation in the mouse hippocampus. n = 6. Unpaired t-test. **E** Representative confocal images of colocalization of MAP2 (red), FITC-PNA (green), C1q (gray), and nuclei (blue) in the CA1 region of the two groups. Scale bars = 20 μm, Scale bars = 10 μm. **F** Quantitative real-time PCR analysis of Neu1, Neu2, Neu3, and Neu4 mRNA expression in the mouse hippocampus. n = 4. Unpaired t-test. **G** Representative confocal images of colocalization of MAP2 (red), FITC-PNA (green), and C1q (gray) in the CA1 region in control and sevoflurane-treated mice that received PBS or NADNA. Scale bars = 20 μm, Scale bars = 10 μm. **H** Quantitative analysis showed that NADNA decreased desialylation in the hippocampus of sevoflurane-treated mice. n = 6. One-way ANOVA followed by a post hoc Tukey’s test. **I** Linear regression analysis showed that neuronal desialylation was significantly related to the colocalization of C1q with MAP2. Data are mean ± SEM. *P < 0.05, ** P < 0.01, *** P < 0.001

The neuronal cell surface is characterized by a high density of sialic acid residues on glycoproteins and glycolipids. Removal of sialic acids, termed desialylation, promotes the binding ability of C1q, which plays a crucial role in opsonization and phagocytosis [31]. We confirmed the presence of sialic acids using PSA-NCAM and detected the loss of sialic acids using FITC-PNA. Sevoflurane significantly suppressed PSA-NCAM expression in the CA1 region (*P* < 0.001) (Fig. 5A, B) and increased the intensity of FITC-PNA (*P* < 0.001) (Fig. 5C, D), suggesting that sevoflurane promoted desialylation on the CA1 neuronal cell surface. The colocalization of MAP2 (a marker of neuronal cell bodies and dendrites), FITC-PNA, and C1q showed that the increased expression of C1q was accompanied by desialylation in the CA1 neurons in the sevoflurane-treated mice (Fig. 5E).

In mammals, sialic acid residues in sialylglycoconjugates are cleaved by sialidases such as Neu1, Neu2, Neu3, and Neu4, which serve as regulators of the sialylation level of glycans on neurons [32]. We found that the mRNA expression of Neu1, Neu2, and Neu4 was increased after sevoflurane exposures (Fig. 5F). Thus, we further investigated the effects of inhibiting the activity of sialidase with NADNA on desialylation and C1q binding to synapses (Fig. 5G). NADNA significantly inhibited the levels of PNA in the CA1 region of sevoflurane-treated mice (*P* < 0.001) (Fig. 5H) and increased the level of PSA-NCAM (*P* < 0.001) (Additional file 1: Fig. S8A, B). Furthermore, we performed a linear regression to confirm that the degree of neuronal desialylation (PNA positive area) was significantly associated with the colocalization of C1q and MAP2 (r^2^ = 0.8979, *P* < 0.001) (Fig. 5I). NADNA also markedly reduced the colocalization of C1q and PSD95 in the CA1 region of sevoflurane-treated mice (*P* = 0.012) (Additional file 1: Fig. S8C, D).

Taken together, these data suggested that neonatal sevoflurane exposures facilitated the loss of neuronal sialic acids and tagging of synapses by C1q through enhancing sialidase activity, which was effectively reversed by NADNA treatment.

### Inhibition of sialidase attenuated sevoflurane-induced cognitive dysfunction by reducing neuronal synapse loss in the hippocampus

**Fig. 6:**
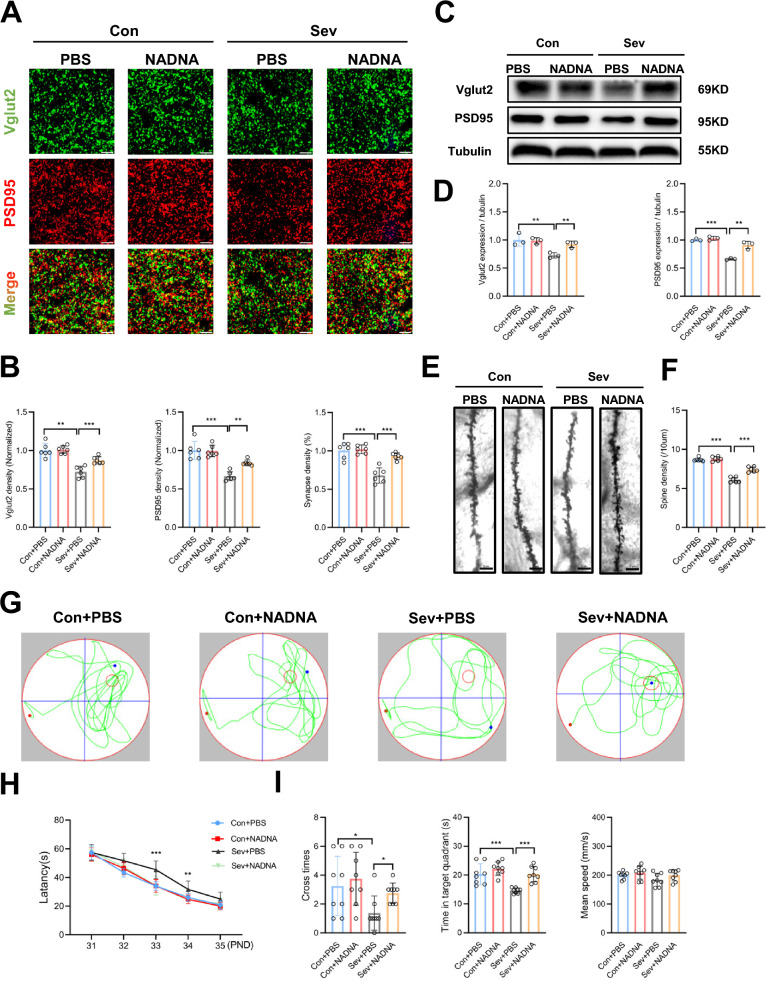
Improvement of sevoflurane-induced cognitive dysfunction by inhibition of sialidase through decreasing neuronal synapse loss. **A** Representative confocal images of Vglut2 (green) and PSD95 (red) in the hippocampus of control and sevoflurane-treated mice with PBS or NADNA treatment. Scale bars = 5 μm. **B** Quantification analysis showed that NADNA treatment increased the density of Vglut2^+^ puncta, PSD95^+^ puncta, and synapses compared to treatment with sevoflurane and PBS. n = 6. One-way ANOVA followed by a post hoc Tukey’s test. **C** Representative Western blot bands of Vglut2 and PSD95 in the four groups. **D** Compared to treatment with sevoflurane and PBS, NADNA up-regulated Vglut2 and PSD95 expression. n = 3. One-way ANOVA followed by a post hoc Tukey’s test. **E** Representative Golgi-Cox staining images of dendrite spines in the four groups. Scale bars = 5 μm. **F** Quantification analysis showed that NADNA treatment increased the spine density compared to treatment with sevoflurane and PBS. n = 6. One-way ANOVA followed by a post hoc Tukey’s test. **G** Tracking plots of mice in the Morris water maze test in the four groups. **H** NADNA treatment reduced the escape latency compared to treatment with sevoflurane and PBS. n = 8. Two-way RM ANOVA followed by a post hoc Tukey’s test. **I** NADNA treatment increased platform cross times and time spent in the target quadrant compared to treatment with sevoflurane and PBS. n = 8. One-way ANOVA followed by a post hoc Tukey’s test. Data are mean ± SEM. *P < 0.05, ** P < 0.01, *** P < 0.001

First, in sevoflurane-treated mice, NADNA administration led to increased density of PSD95 (*P* = 0.002) and Vglut2 (*P* < 0.001) in the hippocampus, as well as increased synapse density (*P* < 0.001) (Fig. 6A, B). NADNA also significantly increased the protein levels of PSD95 and Vglut2 (Fig. 6C, D) and enhanced spine density in Golgi staining analysis (*P* < 0.001) (Fig. 6E, F). Then, we detected the effect of NADNA on microglial synaptic engulfment. The results showed that NADNA significantly decreased microglial-mediated synapse engulfment (*P* < 0.001) in the hippocampus of sevoflurane-treated mice (Additional file 1: Fig. S9A, B). These data indicated that the inhibition of sialidase activity alleviated synapse loss induced by sevoflurane.

We ruled out possible damage to vision and swimming ability due to sevoflurane or NADNA that may interfere with the MWM test (Additional file 1: Fig. S10A, B). Finally, we assessed the effect of inhibiting sialidase activity on neurocognitive deficits in mice induced by repeated sevoflurane exposures (Fig. 6G). In sevoflurane-treated mice, NADNA administration significantly shortened the escape latency on PNDs 33–34 compared with PBS (34.31 ± 6.57 vs. 45.4 ± 6.16 s, *P* < 0.001 on day 33; 24.38 ± 2.59 vs. 31.71 ± 5.76 s, *P* = 0.003 on day 34) (Fig. 6H). Mice treated with NADNA also had increased platform crossing times (2.75 ± 0.71 vs. 1.38 ± 1.19 times, *P* = 0.014) and more time spent in the target quadrant (20.38 ± 2.64 vs. 14.63 ± 0.91 s, *P* < 0.001), without a between-group difference in the mean swimming speed (Fig. 6I).

Taken together, these data suggested that neonatal sevoflurane exposures induced learning and memory impairment through desialylation-mediated synapse loss, and that inhibition of neuronal desialylation by NADNA alleviated the cognitive impairment.

## Discussion

Our results showed that repeated neonatal sevoflurane exposures led to microglial activation, synapse loss, and learning and memory deficits in young mice. Inhibition of microglial activation and phagocytosis with minocycline reduced synapse loss in mice treated with sevoflurane. We then investigated the role of C1q in synapse loss and found that microglia activated by sevoflurane exposures mainly engulfed the C1q-binding synapses. The inhibition of C1q expression with ANX005 suppressed microglial synaptic elimination to improve learning and memory function. We further assessed the involvement of neuronal sialic acids in this process, and the results suggested that enhanced activity of sialidase by sevoflurane exposures mediated the loss of sialic acids, which facilitated C1q binding to synapses. Finally, we inhibited sialidase with NADNA to attenuate desialylation-mediated synapse loss and improve neurocognition.

Sevoflurane-induced neurotoxicity during the neurodevelopment period has gained increasing research interest [33–35]. The brains of newborns are sensitive to anesthetics, and prolonged or repeated sevoflurane inhalation may cause behavioral abnormalities in the long term [36–38]. While synapse loss is one of the main features of sevoflurane-induced neurotoxicity, the underlying molecular mechanism is poorly understood. Microglial-mediated synapse loss has been implicated in neurodegenerative disorders such as Alzheimer’s disease, multiple sclerosis, and frontotemporal dementia [30, 39, 40]. Our data revealed the activation of microglia and their engulfment of synapses in sevoflurane-treated mice.

As both microglia and astrocytes are associated with synapse loss [41, 42], we performed colocalization experiments of PSD95^+^ puncta within IBA1^+^ microglia or GFAP^+^ astrocytes. Our results showed that astrocytes phagocytosed fewer synapses than microglia, suggesting that microglia are the main type of glia mediating synaptic engulfment. Microglia phagocytose synapses by recognizing receptors different from those recognized by astrocytes [43, 44]. During early postnatal development, astrocytes rapidly polarized and engulfed numerous small dendritic apoptotic bodies, while microglia migrated and engulfed the soma and dendrites [41]. Thus, inhibition of microglial synapse elimination by minocycline rescued synapse loss in sevoflurane-induced developmental neurotoxicity.

During early development, superfluous synapses are eliminated by microglia via C1q-mediated synapse pruning to maintain brain homeostasis [45, 46]. By binding to synapses, C1q activates the complement pathway to increase the expression of C3 and promote C3 tagging to synapses, preparing synapses for microglial phagocytosis through C3 receptors. C1q-dependent synapse elimination has also been identified in various neurodegenerative diseases [47–49]. In Alzheimer’s disease, C1q-mediated microglial engulfment of synapses was inappropriately activated, leading to early synapse loss, which was alleviated by inhibition of C1q with BMS-984923 [50, 51]. Our study demonstrates that C1q was involved in the microglial elimination of synapses in the CA1 region after repeated sevoflurane anesthesia. We also found that C1q was primarily produced by microglia rather than astrocytes, which is in line with recent studies [52–54]. Hence, we suppressed C1q expression using the specific antibody ANX005, which decreased synapse loss and improved memory and learning ability. Moreover, we found that sevoflurane also upregulated the C3 expression, and inhibition of C1q by ANX005 reduced the expression of C3, indicating that C3 served as the downstream of C1q.

Recently, researchers have found that the labeling of C1q relies on certain synaptic signals, specifically the “eat me” and “don’t eat me” signals [24]. The eat-me signals refer to molecules that are either exposed or released from a target cell, which directly induces phagocytosis. Phosphatidylserine is the most widely reported eat-me signal, and its exposure occurs locally at the synapse in the developing CA1 region, promoting synaptic pruning by microglia [55]. Target cells can inhibit phagocytosis using “don’t eat me” signals such as CD47 and sialic acids. Sialic acids are the typical residue of glycolipids and glycoproteins, constituting the main negative charge of the cell surface that inhibits phagocytosis [32]. The removal of sialic acid residues, also known as desialylation, can trigger phagocytosis of cells [56]. Our present study found that repeated sevoflurane exposures induced desialylation of the neuronal cell surface in the CA1 region, whereas the reduction in sialic acid could promote C1q tagging to synapses. We further showed that inhibition of sialidase using NADNA suppressed C1q binding to neuronal synapses, decreased microglia-mediated synapse elimination, increased the density of synapses, and enhanced the number of dendrite spines. In addition, the impairment of learning and memory caused by sevoflurane was also alleviated by NADNA.

Based on recent studies, sevoflurane exposures resulted in long-term functional impairment of neurocognition and hippocampus in rodents. Fan et al. reported that maternal sevoflurane exposure disrupted oligodendrocyte myelination of the hippocampus in offspring on PND 60 [57]. Li et al. reported that neonatal sevoflurane induced hippocampal dendrite spine loss on PND 60 [58]. Jiang et al. reported that sevoflurane treatment on PND 7, 14, and 21 significantly reduced the hippocampal volume in adult mice on PND 97 [59]. Moreover, Zhong et al. found that repeated sevoflurane exposures during postnatal development caused Multiple exposures to sevoflurane across postnatal development may cause cognitive deficits and increased the levels of tau, p-tau, and Aβ in the hippocampus at 18 months [60].

There are several limitations in our study. First, we measured synapse density with PSD95 and Vglut2, which represented excitatory synapses only in the CA1 region. It is unclear whether inhibitory synapses are involved in sevoflurane-induced synaptic loss. Second, the electrophysiology of synapses such as long-term potentiation/depression, EEG changes, or burst suppression was not measured. Previous studies have demonstrated that sevoflurane impaired synapse development and electrophysiological activity [23, 61–64]. Last, we used intraperitoneal injection of ANX005 and NADNA to inhibit the expression of C1q and sialidase respectively, and we did not perform interventions at the gene and transcriptional levels.

## Conclusions

**Fig. 7:**
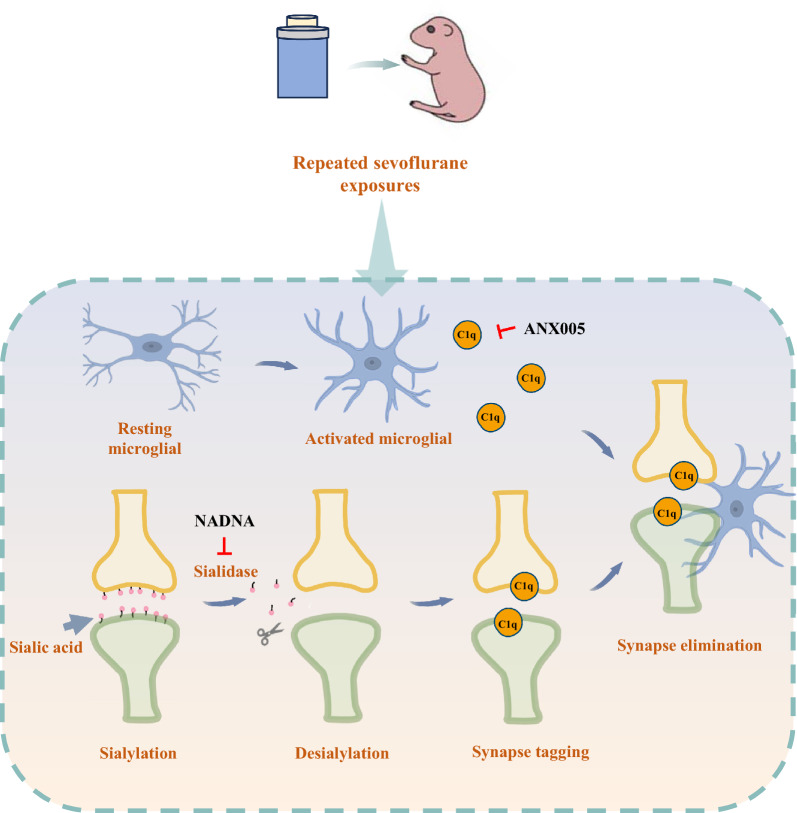
Schematic mechanism of C1q-mediated microglial synaptic elimination via enhanced desialylation underlying developmental neurotoxicity induced by repeated neonatal sevoflurane exposures

Our present study demonstrated that repeated neonatal sevoflurane exposures induced developmental neurotoxicity by activating microglia and aggregating C1q-mediated synaptic loss via neuronal desialylation (Fig. 7). Targeting C1q and sialidase may provide potential therapeutic strategies in the developmental neurotoxicity induced by sevoflurane.

### Supplementary Information

**Additional file 1: Fig. S1.** Neonatal sevoflurane exposures induced activation and phagocytosis of microglia in the mouse hippocampus. (A) Representative confocal images of PSD95^+^ puncta (green), GFAP^+^ astrocytes (gray), and IBA1^+^ microglia (red) in the hippocampus of sevoflurane-treated mice. Scale bars = 20 μm. (B) Quantification analysis showed that the sevoflurane group had increased PSD95^+^ puncta in microglia. n = 6. Unpaired t-test. (C) Representative confocal images of the fluorescent latex beads phagocyted by BV2 cells in two groups. Scale bars = 5 μm. (D) Representative confocal images of IBA1^+^ microglia in the two groups. Scale bars = 20 μm. (E) Quantification analysis showed that sevoflurane led to increased IBA1 intensity. n = 6. Unpaired t-test. (F) Representative confocal images showing the morphology of IBA1^+^ microglia in two groups. Scale bars = 5 μm. (G) Quantification analysis showed that sevoflurane led to increased soma size and reduced total branch points and total process length. n = 20. Unpaired t-test. **Fig. S2.** Inhibition of microglial phagocytosis with minocycline reduced synapse loss after neonatal sevoflurane exposures. (A) Representative confocal images of IBA1^+^ microglia (red) containing PSD95^+^ puncta (green) in control and sevoflurane-treated mice with PBS or minocycline treatment. Orthographic view and 3D rendering are shown. Scale bars = 5 μm. (B) Quantification analysis showed that minocycline had no impact on the control mice and decreased the engulfment index compared to PBS in the sevoflurane-treated mice. n = 20. One-way ANOVA followed by a post hoc Tukey’s test. (C) Representative confocal images of Vglut2 (green) and PSD95 (red) in the four groups. Scale bars = 5 μm. (D) Quantification analysis showed that minocycline had no impact on the control mice and increased synapse density compared to PBS in the sevoflurane-treated mice. n = 6. One-way ANOVA followed by a post hoc Tukey’s test. **Fig. S3.** Sevoflurane increased C1q expression in BV2 cells. (A) Representative Western blot bands of C1q in BV2 cells in the control and sevoflurane groups. (B) Quantification of Western blot showed that the expression of C1q was increased in sevoflurane-treated BV2 cells. n = 3. Unpaired t-test. **Fig. S4.** C1q was mainly expressed in microglia in the hippocampus of mice exposed to neonatal sevoflurane. (A) Representative confocal images of GFAP^+^ astrocytes (red), IBA1^+^ microglia (red), and C1q (green) in the hippocampus of sevoflurane-treated mice. Scale bars = 20 μm. (B) Colocalization area analysis revealed that C1q was mainly expressed in microglia. n = 6. Unpaired t-test. **Fig. S5.** Sevoflurane increased the protein expression of C3, while inhibition of C1q decreased the levels of C3 in sevoflurane-treated mice. (A) Representative Western blot bands of C3 in the hippocampus of control and sevoflurane-treated mice. (B) Quantification of Western blot showed that the expression of C3 was increased in the hippocampus of sevoflurane-treated mice. n = 3. Unpaired t-test. (C) Representative Western blot bands of C3 in the hippocampus of control and sevoflurane-treated mice with IgG or anti-C1q (ANX005) treatment. (D) Compared to treatment with sevoflurane and IgG, ANX005 downregulated C3 expression. n = 3. One-way ANOVA followed by a post hoc Tukey’s test. **Fig. S6.** Inhibition of C1q by ANX005 decreased the phagocytosis of BV2 cells after sevoflurane treatment. Representative confocal images of the fluorescent latex beads phagocyted by BV2 cells in control and sevoflurane-treated BV2 cells with IgG or ANX005 treatment. Scale bars = 5 μm. **Fig. S7.** Sevoflurane or ANX005 treatment did not affect the vision or swimming ability of mice. (A-B) The visible platform trial results showed no differences among groups in time to find the platform and swimming speed. n = 8. One-way ANOVA followed by a post hoc Tukey’s test. **Fig. S8.** Inhibition of sialidase by NADNA decreased C1q tagging to synapses after neonatal sevoflurane exposures. (A) Representative confocal images of PSA-NCAM in the CA1 region of control and sevoflurane-treated mice with PBS or NADNA treatment. Scale bars = 100 μm, Scale bars = 20 μm. (B) Quantitative analysis of PSA-NCAM immunofluorescence indicated that NADNA increased PSA-NCAM in sevoflurane-treated mice. n = 6. One-way ANOVA followed by a post hoc Tukey’s test. (C) Representative confocal images of colocalization of C1q (green) and PSD95 (red) in the four groups. Scale bars = 5 μm. (D) Quantitative analysis of C1q and PSD95 colocalization revealed that NADNA reduced C1q tagging to synapses in sevoflurane-treated mice. n = 6. One-way ANOVA followed by a post hoc Tukey’s test. **Fig. S9.** Inhibition of sialidase by NADNA decreased microglial-mediated synapse engulfment after neonatal sevoflurane exposures. (A) Representative confocal images of IBA1^+^ (red) containing PSD95^+^ puncta (green) in the hippocampus of control and sevoflurane-treated mice with PBS or NADNA treatment. Orthographic view and 3D rendering are shown. Scale bars = 5 μm. (B) Quantification analysis showed that NADNA treatment decreased the engulfment index compared to treatment with sevoflurane and PBS. n = 20. One-way ANOVA followed by a post hoc Tukey’s test. **Fig. S10.** Sevoflurane or NADNA treatment did not affect the vision or swimming ability of mice. (A-B) The visible platform trial results showed no differences among groups in time to find the platform and swimming speed. n = 8. One-way ANOVA followed by a post hoc Tukey’s test.

**Additional file 2: Table S1.** Characteristics of weight, blood gas, and electrolytes of mice.

## Data Availability

The datasets used and/or analyzed during the current study are available from the corresponding author on reasonable request.

## Abbreviations

DEGs: Differentially expressed genes
FBS: Fetal bovine serum
MWM: Morris water maze
NADNA: N-acetyl-2,3-dehydro-2-deoxyneuraminic acid
NCAM: Neural cell adhesion molecule
PBS: Phosphate-buffered saline
PNA: Peanut agglutinin
PND: Postnatal day
PSA: Polysialic acid
RT-qPCR: Real-time quantitative polymerase chain reaction
SEM: Standard error of the mean
SDS-PAGE: Sodium dodecyl sulfate–polyacrylamide gel electrophoresis
PVDF: Polyvinylidene fluoride
TBST: Tris-buffered saline and tween
FDR: False discovery rate
FC: Fold change
