# Supplementary figures and images for: Complement C1q-mediated microglial synaptic elimination by enhancing desialylation underlies sevoflurane-induced developmental neurotoxicity
Source: Cell Biosci. 2024 Apr 1;14:42. doi: 10.1186/s13578-024-01223-7 (PMC10983687; doi:10.1186/s13578-024-01223-7)

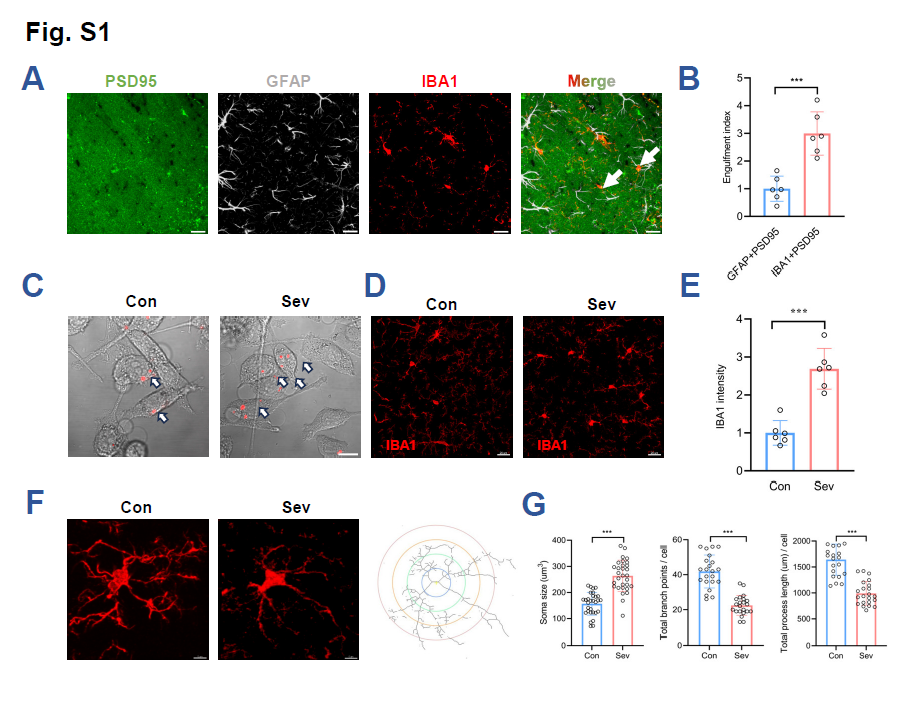


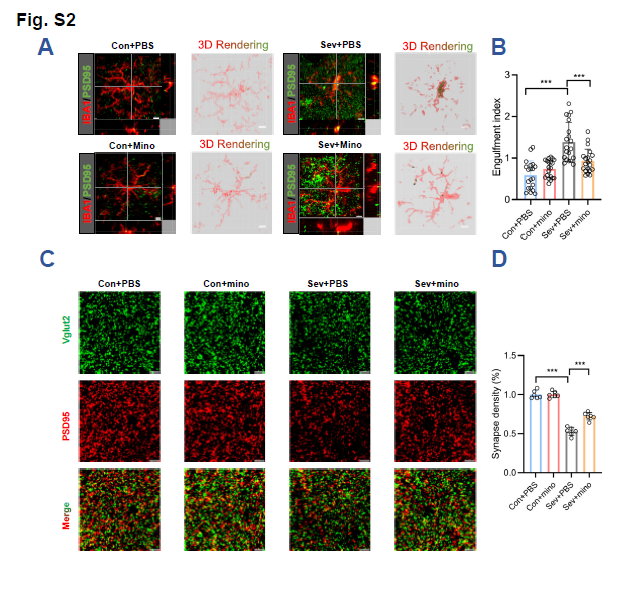


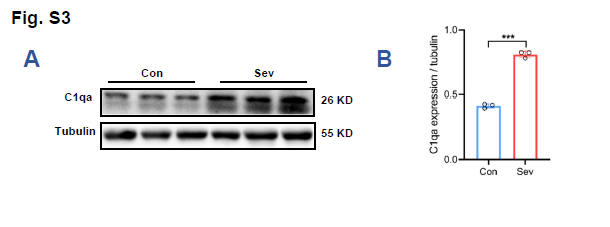


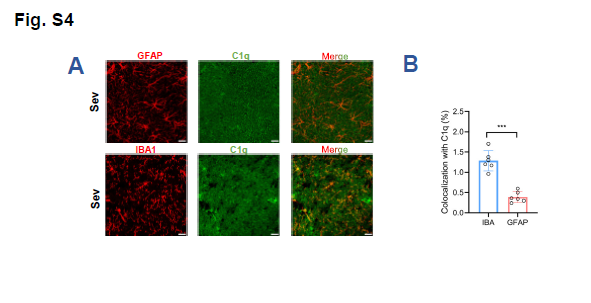


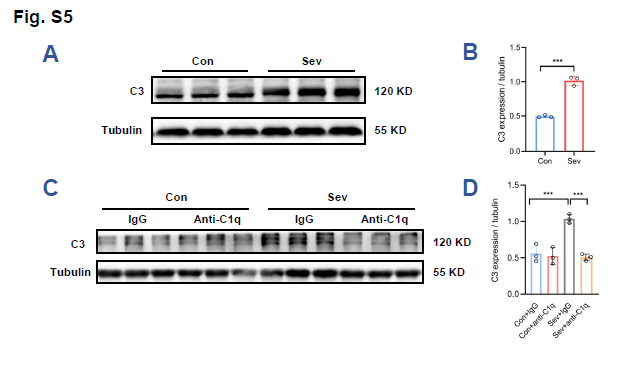


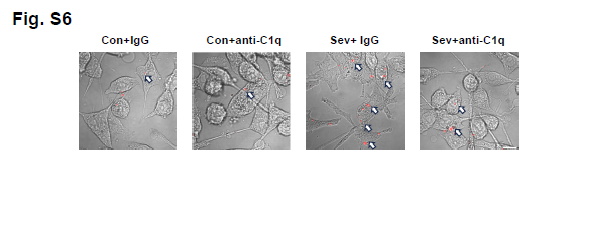


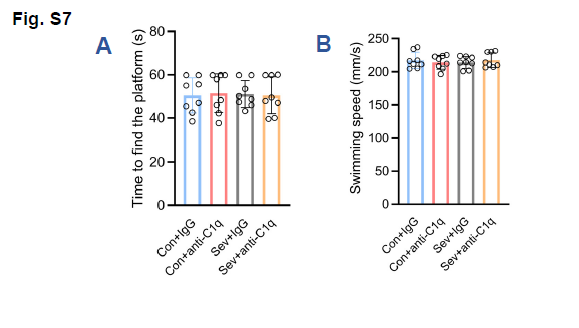


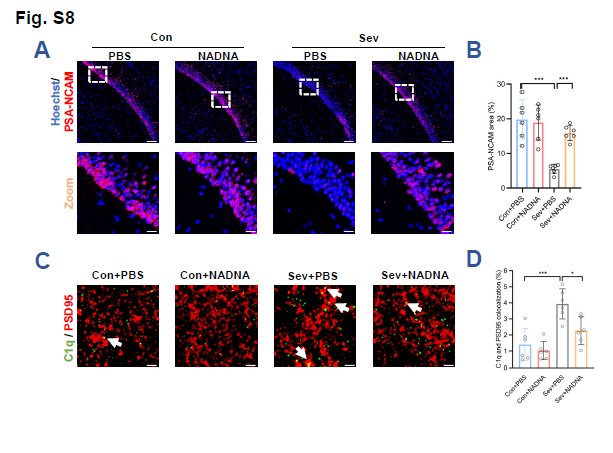


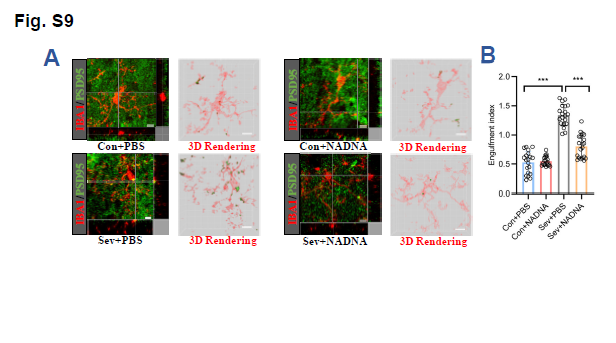


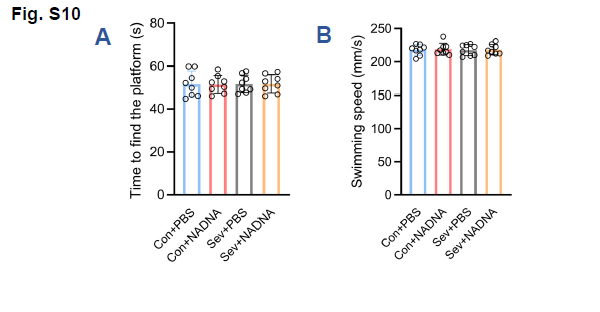

Supplement: Supplementary file 1 — Additional file 1: Fig. S1. Neonatal sevoflurane exposures induced activation and phagocytosis of microglia in the mouse hippocampus. (A) Representative confocal images of PSD95+ puncta (green), GFAP+ astrocytes (gray), and IBA1+ microglia (red) in the hippocampus of sevoflurane-treated mice. Scale bars = 20 μm. (B) Quantification analysis showed that the sevoflurane group had increased PSD95+ puncta in microglia. n = 6. Unpaired t-test. (C) Representative confocal images of the fluorescent latex beads phagocyted by BV2 cells in two groups. Scale bars = 5 μm. (D) Representative confocal images of IBA1+ microglia in the two groups. Scale bars = 20 μm. (E) Quantification analysis showed that sevoflurane led to increased IBA1 intensity. n = 6. Unpaired t-test. (F) Representative confocal images showing the morphology of IBA1+ microglia in two groups. Scale bars = 5 μm. (G) Quantification analysis showed that sevoflurane led to increased soma size and reduced total branch points and total process length. n = 20. Unpaired t-test. Fig. S2. Inhibition of microglial phagocytosis with minocycline reduced synapse loss after neonatal sevoflurane exposures. (A) Representative confocal images of IBA1+ microglia (red) containing PSD95+ puncta (green) in control and sevoflurane-treated mice with PBS or minocycline treatment. Orthographic view and 3D rendering are shown. Scale bars = 5 μm. (B) Quantification analysis showed that minocycline had no impact on the control mice and decreased the engulfment index compared to PBS in the sevoflurane-treated mice. n = 20. One-way ANOVA followed by a post hoc Tukey’s test. (C) Representative confocal images of Vglut2 (green) and PSD95 (red) in the four groups. Scale bars = 5 μm. (D) Quantification analysis showed that minocycline had no impact on the control mice and increased synapse density compared to PBS in the sevoflurane-treated mice. n = 6. One-way ANOVA followed by a post hoc Tukey’s test. Fig. S3. Sevoflurane increased C1q expressi [file 13578_2024_1223_MOESM1_ESM.docx]
