# Supplementary material for: Complement C1q-mediated microglial synaptic elimination by enhancing desialylation underlies sevoflurane-induced developmental neurotoxicity
Source: Cell Biosci. 2024 Apr 1;14:42. doi: 10.1186/s13578-024-01223-7 (PMC10983687; doi:10.1186/s13578-024-01223-7)
Supplement: Supplementary file 2 — Additional file 2: Table S1. Characteristics of weight, blood gas, and electrolytes of mice. [file 13578_2024_1223_MOESM2_ESM.docx]

**Table S1.** Characteristics of weight, blood gas, and electrolytes of mice

|  | **Con (n = 8)** | **Sev (n = 8)** | ***P* value** |
| --- | --- | --- | --- |
| **Weight (g)** | 22.03 ± 1.23 | 22.14 ± 1.05 | 0.96 |
| **PO_2_ (mmHg)** | 97.63 ± 6.52 | 94.57 ± 7.34 | 0.89 |
| **PCO_2_ (mmHg)** | 37.05 ± 5.43 | 38.72 ± 6.01 | 0.93 |
| **Hct (%)** | 38.79 ± 4.27 | 37.91 ± 5.18 | 0.97 |
| **pH** | 7.36 ± 1.22 | 7.31 ± 1.67 | 0.99 |
| **Na^+^ (mmol/L)** | 146.21 ± 0.97 | 145.97 ± 0.86 | 0.97 |
| **Ca^2+^ (mmol/L)** | 1.25 ± 0.52 | 1.31 ± 0.69 | 0.90 |
| **K^+^ (mmol/L)** | 3.84 ± 0.82 | 3.93 ± 0.87 | 0.87 |
